# Head and Neck Cancer Stem Cell-Enriched Spheroid Model for Anticancer Compound Screening

**DOI:** 10.3390/cells9071707

**Published:** 2020-07-16

**Authors:** Larisa Goričan, Boris Gole, Uroš Potočnik

**Affiliations:** 1 Centre for Human Molecular Genetics and Pharmacogenomics, Faculty of Medicine, University of Maribor, SI-2000 Maribor, Slovenia; larisa.gorican@um.si (L.G.); boris.gole@um.si (B.G.); 2 Laboratory for Biochemistry, Molecular biology and Genomics, Faculty of Chemistry and Chemical Engineering, University of Maribor, SI-2000 Maribor, Slovenia

**Keywords:** head and neck squamous cell carcinoma, cancer stem cells, 3-D/spheroid culture/model, high-throughput screening, anticancer stem cell compounds, ATRA, stem cell marker

## Abstract

Cancer stem cells (CSCs), a rare cell population in tumors, are resistant to conventional chemotherapy and thus responsible for tumor recurrence. To screen for active compounds targeting CSCs, a good CSC-enriched model compatible with high-throughput screening (HTS) is needed. Here, we describe a new head and neck cancer stem cell-enriched spheroid model (SCESM) suitable for HTS analyses of anti-CSC compounds. We used FaDu cells, round-bottom ultra-low adherent (ULA) microplates, and stem medium. The formed spheroids displayed increased expression of all stem markers tested (qRT-PCR and protein analysis) in comparison to the FaDu cells grown in a standard adherent culture or in a well-known HTS-compatible multi-cellular tumor spheroid model (MCTS). Consistent with increased stemness of the cells in the spheroid, confocal microscopy detected fast proliferating cells only at the outer rim of the SCESM spheroids, with poorly/non-proliferating cells deeper in. To confirm the sensitivity of our model, we used ATRA treatment, which strongly reduced the expression of selected stem markers. Altogether, we developed a CSC-enriched spheroid model with a simple protocol, a microplate format compatible with multimodal detection systems, and a high detection signal, making it suitable for anti-CSC compounds’ HTS.

## 1. Introduction

Head and neck squamous cell carcinoma (HNSCC) is one of the most common malignancies worldwide with a ~50% five-year survival rate. Mortality is mainly attributed to local recurrences, cervical lymph node metastases, and occasionally distant organ metastases [1,2]. HNSCC growth is maintained by a population of cancer stem cells (CSCs) characterized by a dormant state with infrequent divisions, resistance to conventional antitumor therapies, and ability to activate, multiply, and differentiate after therapy [3,4,5,6]. CSCs’ resistance to therapy is thus a major impediment to successful treatment, making effective targeting of the CSCs essential for complete tumor eradication. [7].

However, since CSCs are a rare population within tumors, they are difficult to isolate and examine. Thus, to assist the search for biologically active compounds with anti-CSCs activity, CSCs enrichment models are being developed [8,9,10]. CSCs can mainly be obtained from cancer cell lines or primary tumors through reprogramming, selection of cells resistant to anoikis (the so-called culture of free-floating spheres, i.e., tumorsphere model and spheroid forming assay), or based on the application of specific culture conditions (i.e., multi-cellular tumor spheroid model, MCTS, among others) [8,11].

Among the most commonly used spheroid forming techniques in HNSCC are cultures of free-floating spheres and MCTS [12,13,14,15,16]. Cultures of free-floating spheres are technically quite challenging as they demand long-term cultivation (2–3 weeks), multi-step cell culture manipulations, and growing cells on flat low-adherent surfaces, causing the generation of multiple non-uniform spheroids with no spatial separation, which limits the use of this model for compound screenings [17]. On the other hand, MCTS is based on high-density microplate formats, simple protocols, and is compatible with automated and multimodal detection systems [18]. Unfortunately, the method only partially enriches for CSCs, since it uses serum-supplemented cell culture media, which were recently shown to promote cellular differentiation and hinder CSCs expansion [12,19,20,21,22].

Here, we combined features of the two most commonly used HNSCC spheroid-forming techniques and developed a new stem cell-enriched spheroid model (SCESM) adaptable for high-throughput screenings (HTSs) of anti-CSC compounds. The SCESM uses a commercial HNSCC cell line, proper stem cell medium, and round-bottom ULA plates, generating a single uniform spheroid per well. It is characterized by simple procedures, short cultivating time, compatibility with high-density microplate formats, and exhibits CSCs enrichment much higher than that observed in the MCTS model. Additionally, we treated SCESM with all-trans retinoic acid (ATRA), a known differentiating agent used also in HNSCC [23,24], which resulted in strongly reduced stem marker expression, thereby validating SCESM’s sensitivity for anti-CSCs active substances screening.

## 2. Materials and Methods

### 2.1. Cells and Monolayer Cell Culture

The FaDu HNSCC cell line was obtained from American Type Culture Collection (ATCC, Manassas, VA, USA). The cells were cultured in 1:1 mixture of Dulbecco’s Modified Essential Medium and Ham’s F-12 Medium (DMEM/F12, GibcoTM ThermoFisher Scientific, Waltham, MA, USA), supplemented with 10% FBS (GibcoTM) and 1% penicillin/streptomycin (P/S) (Sigma-Aldrich, St. Louis, MO, USA) and maintained under standard culture conditions (37 °C, %5 CO_2_, >90% relative humidity).

### 2.2. MCTS Cell Culture

FaDu MCTS were generated as previously published [25]. Briefly, for the generation of FaDu MCTSs in 96-well round bottom ULA plates (Cat. No. 7007; Corning, NY, USA), FaDu cells were resuspended in DMEM/F12 medium (GibcoTM), supplemented with 10% FBS (GibcoTM) and 1% P/S (Sigma-Aldrich). In total, 200 μL of FaDu single-cell suspension with a concentration of 3500 cells per well were transferred into each well. ULA plates were centrifuged at 710× *g* for 10 min and then kept under standard culture conditions for seven days with a half medium change every two to three days.

### 2.3. SCESM Cell Culture

For the generation of SCESM spheroids, two previously published protocols were combined and modified [25,26]. FaDu cells were resuspended in stem medium composed of DMEM/F12 medium (GibcoTM) supplemented with 10 ng/mL of epidermal growth factor (ThermoFisher Scientific), 10 ng/mL of basic fibroblast growth factor (ThermoFisher Scientific), B-27^TM^ (50×) serum-free supplement (ThermoFisher Scientific), and 1% P/S (Sigma-Aldrich), and seeded in 96-well round-bottom ULA plates (Corning) at concentrations of 1500, 2500, or 3500 cells/well. ULA plates were centrifuged at 710× *g* for 10 min and then cultured under standard culture conditions for seven days with a half medium change every two to three days.

### 2.4. Measurement of Spheroid Size

The spheroid size was analyzed by using a bright-field Axiovert 40 microscope (Zeiss, Oberkochen, Germany) and photos were captured with a Zeiss Axiocam 506 camera (Zeiss). The spheroid mean diameter was determined by using FiJi software v. 1.51 (Fiji.sc/Fiji).

### 2.5. ATRA Treatment

Seven-day-old SCESM spheroids were further cultured in FaDu standard growth medium or stem medium and treated with 10 μM ATRA (Sigma-Aldrich) for two or five days under standard culture conditions. In case of the five-day treatment, half medium was exchanged on day three.

### 2.6. Confocal Microscopy

To analyze whole SCESM spheroids, seven-day-old spheroids were fixed using 3.7% formaldehyde (Sigma-Aldrich) in D-PBS (Sigma-Aldrich) for 30 min at room temperature (RT). Next, the spheroids were permeabilized with 0.5% triton X-100 (Sigma-Aldrich) overnight at 4 °C and blocked with 1% FBS (GibcoTM) in D-PBS (Sigma-Aldrich) at RT for 30 min. Spheroids were then labeled with Alexa Fluor^®^ 488-conjugated mouse anti-human Ki-67 (#A4-155-T100, ExBio, Prague, Czech Republic) at 4 °C overnight. Next, the nuclei were stained using 7-amino-actinomycin D (7-AAD, #00-6993-50, Invitrogen, ThermoFisher Scientific) (2.5 µg/mL) and immediately analyzed with a microscope.

One image per spheroid at each wavelength (focused on the spheroid center) was captured by a Leica TCS SP5-II one photon inverted confocal microscope (Leica, Wetzlar, Germany) with a 10× objective.

The anti-Ki-67 antibody was excited with a 488-nm laser line from an argon laser, and 7-AAD was excited with a 561-nm laser line from a DPSS 561 laser. For the visualization of Ki-67, the emission window was set at 485–565 nm and for visualization of 7-AAD, the emission window was set at 607–697 nm.

For each spheroid analyzed, separate photos for fluorescent antibody and 7-AAD were captured. Using FiJi software v. 1.51 (Fiji.sc/Fiji), we constructed a graph of the fluorescence intensity according to the spheroid diameter.

### 2.7. mRNA Analysis

Total RNA was extracted using Trizol (ThermoFisher Scientific) and reverse transcribed using a High-Capacity cDNA Reverse Transcription Kit (Applied Biosystems™ ThermoFisher Scientific). The synthesized cDNA was used to perform qPCR gene expression analysis for selected genes, using a QuantStudio 12K Flex Real-Time PCR System (ThermoFisher Scientific). The primers are listed in Table 1. The relative expression of the target genes was normalized against Beta-2-Microglobulin gene (*B2M*). Melting curves were examined with regard to the quality of PCR amplification for each sample and quantification was performed using the comparative Ct (2^−ΔΔCt^) method.

### 2.8. Flow Cytometry

For flow cytometry analysis, the 2-D and 3-D cancer cells were trypsinized using 0.25% EDTA trypsin (GibcoTM). The cells were fixed with 3.7% formaldehyde (Sigma-Aldrich) in D-PBS (Sigma-Aldrich) at RT for 5 min and blocked with 1% FBS (GibcoTM) in D-PBS at RT for 10 min. Cells were then labeled overnight at 4 °C with primary monoclonal antibodies: PerCP-Cyanine5.5-conjugated rat anti-human CD44 (#45-0441-82, ThermoFisher Scientific), PE-conjugated mouse anti-human CD73 (#1P-675-T100, ExBio), PE-conjugated mouse anti-human CD133 (#1P-819-T100, ExBio), and mouse anti-human CD90 (#sc-53456, SCBT, Dallas, TX, USA). Since mouse anti-human CD90 was not conjugated with a fluorophore, these samples were, after overnight incubation, stained with PerCPeFluor 710-conjugated goat anti-mouse IgG secondary antibody (#46-4010-82, ThermoFisher Scientific) for 1 h at room temperature. Flow cytometry was performed on a Luminex Image Stream X MK II System (Luminex Corporation, Austin, TX, USA) and data analysis was completed by Image Stream software Ideas v 6.2 (Luminex Corporation). Each experiment was performed 5 times (*n* = 5) with more than 2000 events (*N* > 2000) measured each time.

### 2.9. Western Blot

For Western blot, we used a previously published protocol [27]. Briefly, cells were lysed in RIPA buffer (50 mMTris/HCl, pH 7.4, 150 mM NaCl, 2 mM EGTA, 2 mM EDTA, 25 mM NaF, 25 mM β-glycerophosphate, 0.1 mM NaV, 0.2% Triton X-100, 0.3% Nonidet P40) supplemented with proteinase inhibitors (Sigma-Aldrich). Total protein concentration was measured by the Bradford method using bovine serum albumin (BSA) as the standard. Proteins were loaded onto 15% SDS–PAGE gels and electrophoresed using an XCell SureLock Electrophoresis System (ThermoFisher Scientific). The protein samples were transferred to a nitrocellulose membrane using iBlot, a dry blot gel transfer device (ThermoFisher Scientific).

Next, the membrane was blocked with 5% dried skimmed milk powder for 2 h at room temperature. The membranes were incubated with rabbit anti-human POU5F1 (# MA5-14845, ThermoFisher Scientific), mouse anti-human alpha-tubulin (#ab7291, Abcam, Cambridge, UK), and mouse anti-human involucrin (#MA5 11803, ThermoFisher Scientific) primary antibodies at 4 °C overnight. Then, the HRP-conjugated goat anti-rabbit (#ab97080, Abcam) and HRP-conjugated goat anti-mouse (#ab205719, Abcam) secondary antibodies were used. Western blot signals were visualized by Clarity Western ECL Substrate (Bio-Rad Laboratories, Hercules, CA, USA) with a G:Box imaging system (Syngene, UK) and quantified using Image Lab software v. 6.0.1 (Bio-Rad Laboratories).

### 2.10. Statistical Analysis

All data are presented as the mean ± standard error (SE). To test for data distribution, the D’Agostino–Pearson omnibus normality test was used. The gene expression data showed an abnormal distribution, hence for assessment of the statistical significance between two groups, the nonparametric Mann–Whitney test was used. The protein expression data also showed an abnormal distribution, hence for assessment of the statistical significance between two groups, the nonparametric Kruskal–Wallis ANOVA test was used followed by ad hoc Dunn’s multiple comparisons test. Statistical analyses were performed using GraphPad Prism software v. 8 (GraphPad Software Inc., San Diego, CA, USA). * *p* < 0.05, ** *p* < 0.01, *** *p* < 0.001, or **** *p* < 0.0001 were considered statistically significant. Individual experiments were independently repeated at least three times.

## 3. Results

### 3.1. Defining SCESM Growth Conditions

**Figure 1:**
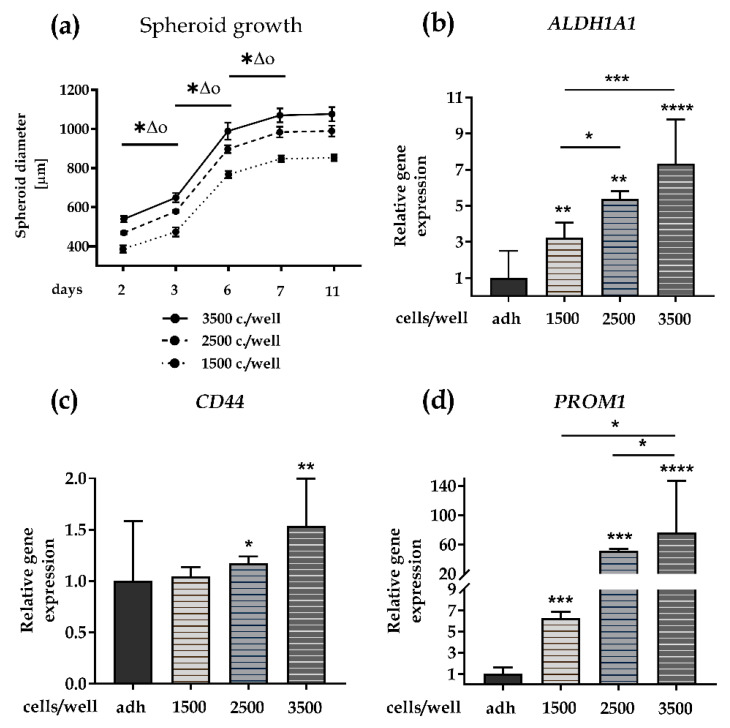
Stem cell-enriched spheroid model (SCESM) spheroid growth condition analysis. Growth of SCESM spheroids with three different cell seeding concentrations, i.e., 3500, 2500, and 1500 cells per well, was analyzed and relative gene expression of head and neck squamous cell carcinoma (HNSCC) stem markers was assessed; (**a**) Growth curve of SCESM spheroids cultured for 11 days. Mean values ± SE (*n* = 4) are presented, (*, ∆, and o represent *p* < 0.05, * is used for comparisons in group 1500 c./well, ∆ is used for comparisons in group 2500 c./well, o is used for comparisons in group 3500 c./well); (**b**–**d**) Relative gene expression of stem markers in 7-day-old SCESM spheroids and an adherent cell line. Normalized mean values ± SE (*n* ≥ 4) are presented. Asterisks above the graph bars represent the results of statistical analysis of the studied group vs. the control group—adh (* *p* < 0.05, ** *p* < 0.01, *** *p* < 0.001, **** *p* < 0.0001); (**b**) Relative gene expression of *ALDH1A1*; (**c**) Relative gene expression of *CD44*; (**d**) Relative gene expression of *PROM1*. adh, adherent cell line.

As the first step in designing SCESM, we determined optimal growth conditions enabling high CSC enrichment in a short time. We seeded FaDu cells, which were previously shown to express HNSCC stem markers and form compact spheroids [28], in stem cell medium in 96-well ULA plates at 1500, 2500, and 3500 cells per well and monitored the spheroid growth for 11 days, while changing the medium every other day. Regardless of the cell number seeded, compact spheroids formed within two days and, as expected, the average spheroid diameter reflected the initial seeding numbers, with 3500 seeded cells yielding the biggest spheroids at all time points (Figure 1a). From the start, all the spheroids increased in size significantly (8.2–62.0%, *p* < 0.05) between the two consecutive measurements, i.e., day 2 and 3, 3 and 6, and 6 and 7.

Interestingly though, independently of the seeding cell concentration, spheroid growth reached a plateau on day 7 (850, 1000, and 1100 μm diameter for 1500, 2500, and 3500 seeded cells, respectively), with no significant diameter change until day 11 (*p* = 0.49). As the spheroids from 3500 seeded cells from day 6 onward depleted the medium within 2 days (noticeable change in color of the media, not shown), we did not try seeding higher cell numbers. Of note, all of the spheroids exceeded the 500 μm diameter, which enables the development of hypoxia in the spheroid core, thus promoting CSC enrichment, already on day 3 [28,29,30,31,32].

Since spheroid size seemed to stabilize after 7 days, we used this time point for further tests. First, we analyzed the relative gene expression of three HNSCC stem markers, *ALDH1A1*, *CD44*, and *PROM1* (CD133) [33], in seven-day-old spheroids. With the exception of *CD44* in spheroids from 1500 seeded cells, the expression of all three markers was significantly increased in all of the spheroids compared to the FaDu cells grown in the adherent monolayer culture, consistent with an enhanced content of stem-like cells in the SCESM (Figure 1b–d). The highest increases (7.3-fold, 1.5-fold, and 76.4-fold for *ALDH1A1*, *CD44*, and *PROM1*, respectively) were reached in the spheroids grown from 3500 cells. The spheroids grown from 3500 seeded cells had significantly higher *ALDH1A1*, *CD44*, and *PROM1* expression (2.3-fold and 1.4-fold for *ALDH1A1*; 1.5-fold and 1.3-fold for CD44; 12.2-fold and 1.5-fold for *PROM1*) compared to the spheroids grown from 1500 and 2500 seeded cells, respectively; Figure 1b–d).

These results show that the seeding of 3500 FaDu cells under SCESM conditions for 7 days yields compact spheroids with a stable size of 1100 μm, enabling formation of a CSC-enriched hypoxic core, with significantly increased relative gene expression of HNSCC stem markers. Since our goal was to maximize CSC enrichment in a short time, we chose these conditions for further work with the SCESM model.

### 3.2. Only Surface Cells in the SCESM Are Highly Proliferative

**Figure 2:**
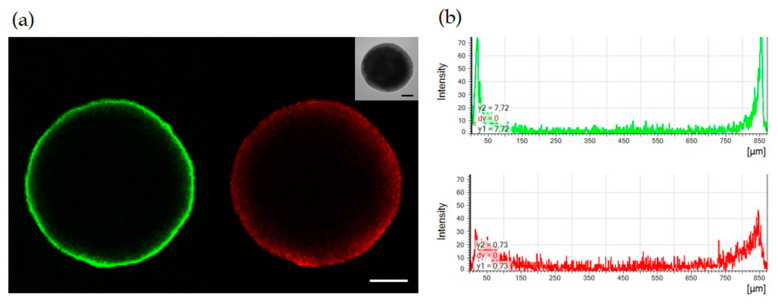
Confocal microscopy analysis of SCESM spheroid. Seven-day-old SCESM spheroid was stained for the presence of the proliferation marker Ki-67 (anti-Ki-67) and spheroid nuclei were stained with 7-amino-actinomycin D (7-AAD). (**a**) Confocal microscope fluorescent and visible light images are presented. Green fluorescence represents Ki-67 and red fluorescence represents 7-AAD staining. Scale bars correspond to 200 μm; (**b**) Histograms of the fluorescence intensity across the SCESM spheroid diameter are presented; the green histogram displays Ki-67 and the red histogram displays the 7-AAD fluorescence intensity across the SCESM spheroid diameter.

As SCESM spheroids’ growth stagnated after 7 days, we assessed their proliferation rate at this time point. To this end, we fixed and permeabilized intact 7-day-old spheroids and stained them for the proliferation marker Ki-67. Confocal microscopy showed an intense Ki-67 signal only on the outer rim of cells, which rapidly decreased within 50 µm of depth and practically disappeared at 100 µm of depth (Figure 2). Counterstaining with 7-AAD nucleic acid stain, on the other hand, revealed the presence of nucleated cells at least to the depth of 150 µm (Figure 2).

This result suggests that fast proliferating cells are present only in the narrow outer rim of the SCESM spheroids, while the cells deeper in are poorly/not proliferating, which is consistent with the enhanced stem-like properties of cells in the SCESM spheroid.

### 3.3. SCESM Expresses High Levels of Stem Markers

As an additional confirmation of the SCESM spheroids’ “stemness”, we measured the relative gene expression of stem markers additional to the HNSCC markers (Figure 1b–d). We chose *POU5F1* (OCT4), *SOX2*, and *NANOG*, all coding for core transcription factors of the human stem cell pluripotency signaling pathway [34,35]; and *THY1* (CD90) and *NT5E* (CD73), which are (together with *CD44*) among the basic human mesenchymal stem cells markers proposed by the International Society for Cellular Therapy [36].

**Figure 3:**
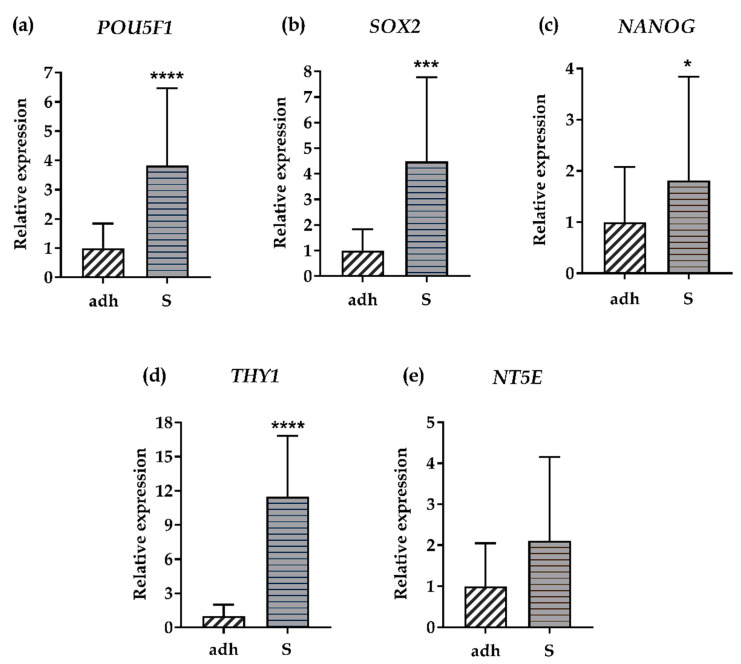
Relative gene expression of stem markers. Relative gene expression of five additional stem markers was assessed in the FaDu adherent cell line and 7-day-old SCESM spheroids. (**a**) Relative gene expression of *POU5F1*; (**b**) Relative gene expression of *SOX2*; (**c**) Relative gene expression of *NANOG*; (**d**) Relative gene expression of *THY1*; (**e**) Relative gene expression of *NT5E*. Normalized mean values ± SE (*n* ≥ 5) are presented (* *p* < 0.05, *** *p* < 0.001, **** *p* < 0.0001 vs. control- adh). adh: adherent cell line; S: SCESM.

The relative gene expression of 4/5 additional stem markers was significantly higher (*POU5F1* 3.8-fold, *SOX2* 4.5-fold, *NANOG* 1.8-fold, and *THY1* 11.5-fold) in SCESM spheroids compared to the adherent cells (Figure 3a–d). *NT5E* expression showed a trend (*p* < 0.1) toward a 2.1-fold increase in spheroids (Figure 3e).

**Figure 4:**
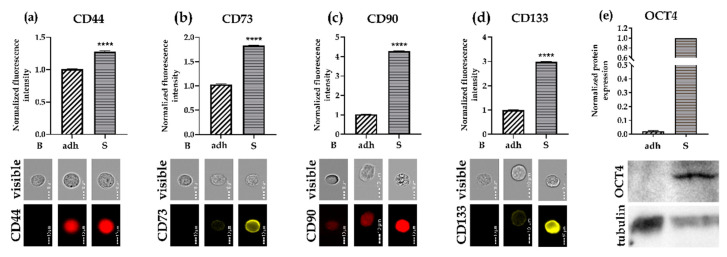
Protein expression of stem markers. The protein expression of five different stem markers was assessed in the FaDu adherent cell line and 7-day-old SCESM spheroids by Western blot and flow cytometry. (**a**–**d**) Protein expression analyzed by flow cytometry. Normalized fluorescence intensities of selected proteins are presented, and corresponding flow cytometry visible light and fluorescent photos are attached. Normalized mean values ± SE (*n* ≥ 5, *N* ≥ 2000) are presented (**** *p* < 0.0001 vs. control (adh)); (**a**) CD44 protein expression; (**b**) CD73 protein expression; (**c**) CD90 protein expression; (**d**) CD133 protein expression; (**e**) OCT4 and tubulin protein expression analyzed by Western blot. Western blot membrane photos showing OCT4 and tubulin and a graph depicting normalized OCT4 protein expression analysis from Western blots are presented. Bars represent normalized mean values ± SE (*n* ≥ 3). B: blank; adh: adherent cell line; S: SCESM.

To further validate the relative gene expression results, we disintegrated 7-day-old spheroids to single cells and checked the protein expression of four surface markers (CD44, CD73, CD90, CD133) using flow cytometry. The results showed significantly increased expression of all the surface markers in the SCESM spheroids compared to the adherent cells (1.3-fold increase of CD44, 1.8-fold increase of CD73, 4.3-fold increase of CD90, and a 3-fold increase of CD133; Figure 4a–d). We also measured the protein expression of one of the transcription markers (OCT4) using Western blot. Again, we observed a 57-fold increase in OCT4 expression in the SCESM spheroids compared to the adherent cells (Figure 4e).

Together, all the tested stem markers confirm that growing FaDu cells under SCESM growth conditions enables significant enrichment of stem-like cells compared to the standard adherent cell culture.

### 3.4. SCESM Grows Better and Expresses Higher Levels of Stem Markers than MCTS

**Figure 5:**
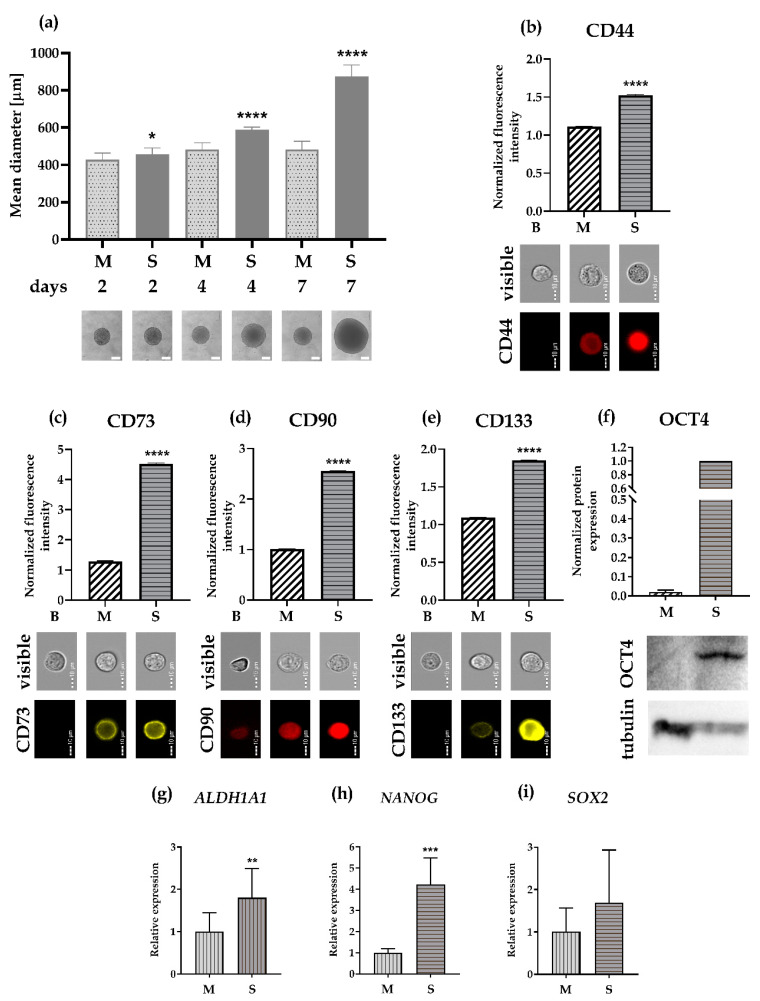
Comparative analysis of SCESM spheroids and multi-cellular tumor spheroids (MCTSs). (**a**) Graph representing SCESM spheroid and MCTS mean diameter increase. Spheroids were photographed on days 2, 4, and 7 and representative phase-contrast microscope images of SCESM spheroids and MCTSs on days 2, 4, and 7 are displayed under the graph. Mean values ± SE (*n* ≥ 8) are presented. Scale bars correspond to 400 µm; (**b**–**e**) Protein expression of four different stem markers was assessed in SCESM spheroids and MCTSs by flow cytometry. Normalized fluorescence intensities of selected proteins are presented and corresponding flow cytometry visible light and fluorescent photos are attached (*n* ≥ 5, *N* ≥ 2000); (b) CD44 protein expression; (**c**) CD73 protein expression; (**d**) CD90 protein expression; (**e**) CD133 protein expression; (**f**) OCT4 and tubulin protein expressions were assessed in SCESM spheroids and MCTSs by Western blot analysis. Western blot membrane photo showing OCT4 and tubulin and graph depicting normalized OCT4 protein expression analysis from Western blots (using Image Lab software) are presented (*n* ≥ 3); (**g**–**i**) Relative gene expression of three additional stem markers assessed in 7-day-old MCTSs and SCESM spheroids; Normalized mean values ± SE (*n* ≥ 5) are presented; (**g**) Relative gene expression of *ALDH1A1*; (**h**) Relative gene expression of *NANOG*; (**i**) Relative gene expression of *SOX2*. (* *p* < 0.05, **** *p* < 0.0001 vs. control (MCTS)). B: blank; M: multi-cellular tumor spheroid; S: SCESM.

Next, we compared our SCESM (using a stem medium) to the conventional MCTS (using medium supplemented only with FBS). To this end, we seeded the same number of cells (3500) for both models. SCESM spheroids displayed a much higher growth rate than the MCTS spheroids, with the average SCESM spheroid diameter being 6.7%, 22%, and 81% larger compared to the MCTS spheroids on days 2, 4, and 7, respectively (Figure 5a). This result shows that SCESM culturing conditions enhance spheroid growth compared to MCTS conditions, resulting in bigger spheroids at the same timepoints.

When the expression of stem cell markers was compared between the two models, flow cytometry clearly showed significantly higher expression of all the surface markers in SCESM than in MCTS (1.4-fold increase of CD44, 3.5-fold increase of CD73, 2.5-fold increase of CD90, and 1.7-fold increase of CD133; Figure 5b–e). On the Western blot, the expression of OCT4 was 47-fold higher in SCESM compared to MCTS (Figure 5f). We also checked the relative gene expression of the three stem markers not tested on the protein level. Here, SCESM had a significantly higher expression of *ALDH1A1* (1.8-fold increase) and *NANOG* (4.2-fold increase) compared to the MCTS, while a 1.6-fold increase of *SOX2* expression was not significant (Figure 5g–i).

Based on these results, SCESM provides larger spheroids (i.e., more material for downstream applications) with cells that are more stem-like than the MCTS model.

### 3.5. ATRA Reduces Stem Marker Expression in SCESM

To confirm the usefulness of SCESM for anti-CSCs compound screening, we tested 7-day-old SCESM spheroids with all-trans retinoic acid (ATRA), a known differentiation compound also used in HNSCC therapy [23,24].

To set an optimal endpoint of the treatment, we treated the spheroids for 2 and 5 days. Since the stem medium used to grow SCESM spheroids contains supplements enhancing the stemness of the cells, which could conceal the ATRA differentiating effect, we used both stem and standard monolayer culture medium (containing 10%FBS, but no other supplements). The five-day ATRA treatment reduced *ALDH1A1* and *CD44* expression compared to the control treatment regardless of the medium used (Supplementary Material Figure S1a–b). Additionally, *PROM1* and *THY1* were also significantly reduced when stem medium was used, while no differences were observed with the *NT5E* and *POUF1* in both media (Supplementary Material Figure S1c–f). Nevertheless, we also observed that if the stem medium was used, the expression of 4/6 tested stem markers increased in the mock-treated spheroids compared to the 7-day-old spheroids (i.e., day 0 of the treatment experiment), leading to the increased differences between the ATRA- and mock-treated spheroids. This is why we decided to select standard medium for further differentiation experiments.

**Figure 6:**
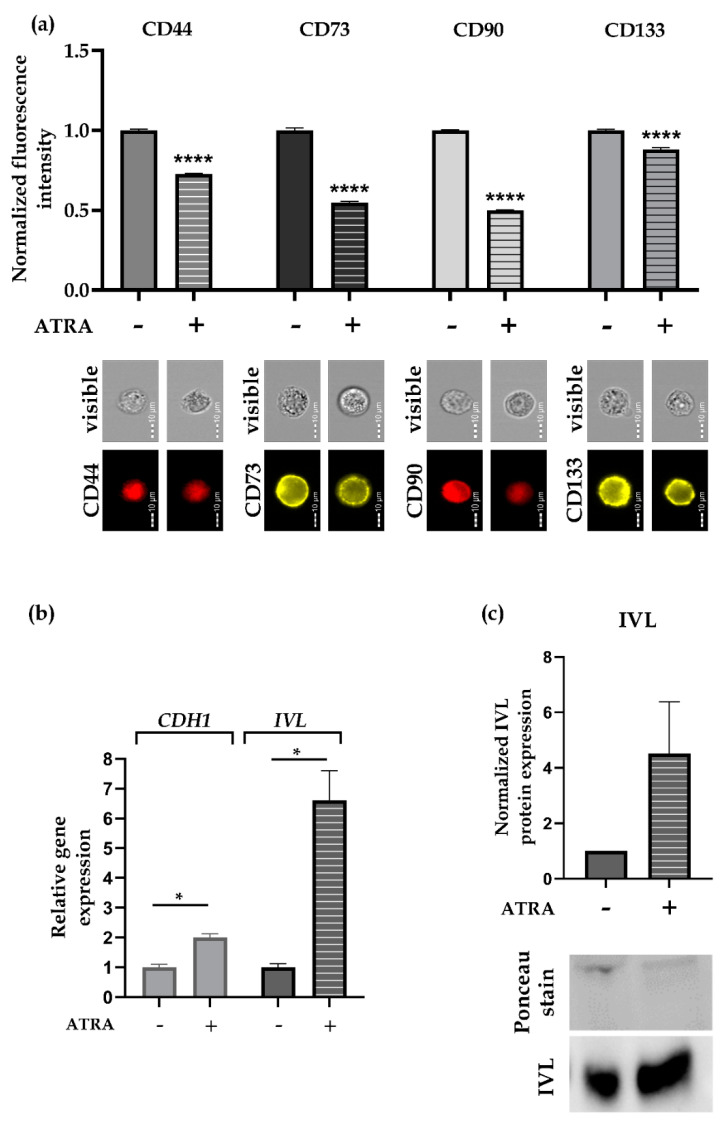
All-trans retinoic acid (ATRA) treatment. Seven-day-old SCESM spheroids were treated with 10 μM ATRA for 5 days. (**a**) Normalized fluorescence intensities of selected surface stem markers and corresponding flow cytometry visible light and fluorescent photos are presented. Normalized mean values ± SE (*n* ≥ 5, *N* ≥ 2000) are shown. (**b**) Relative gene expression of two differentiation markers, *CDH1* and *IVL*, after 5 days of ATRA treatment are displayed. Normalized mean values ± SE (*n* ≥ 5) are presented. (**c**) IVL protein expression was assessed after 5 days of ATRA treatment by Western blot analysis. Western blot membrane photo showing IVL and Ponceau staining and a graph depicting normalized IVL protein expression analysis from the Western blot membrane, (using Image Lab software) are presented (*n* ≥ 3). (* *p* < 0.05, **** *p* < 0.0001 vs. control − ATRA untreated group)—control/ATRA untreated group; +: ATRA treated group.

These showed that 5 days of ATRA treatment reduced the protein levels of all four surface stem markers selected: CD44 by 27%, CD73 by 45%, CD90 by 50%, and CD133 by 12%, compared to the mock-treated SCESM spheroids (Figure 6a), thus validating SCESM’s usefulness for anti-CSCs active substance screening. To further validate our results, we also checked the expression of two epithelial differentiation markers, Cadherin 1 (*CDH1)* and Involucrin (*IVL*) [37,38]. As expected, five days of ATRA treatment significantly increased the mRNA expression levels of both markers (*CDH1* by 2.0-fold and *IVL* by 6.6-fold, *p* < 0.05; Figure 6b). Western blot confirmed the mRNA results for IVL (4.2-fold increase, Figure 6c).

**Figure 7:**
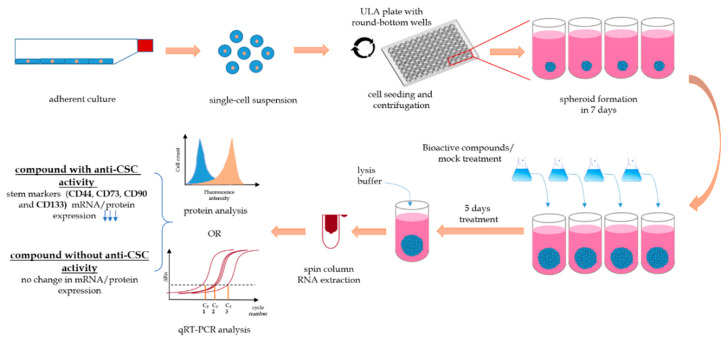
A schematic representation of compound screening with SCESM using relative gene expression and protein expression analysis. A single-cell suspension is seeded with stem medium onto round-bottom ultra-low adherent (ULA) microwell plate and centrifuged to obtain single multi-cellular mass per well. To form spheroids, the cells are cultured for 7 days, with half of the medium changed every 2–3 days. After seven days, half of the stem medium is replaced with treatment medium containing testing compound. Each spheroid can be treated individually with different compounds/concentrations. After five days of treatment, the spheroids are disintegrated by lysis buffer and RNA or protein analysis can be performed. For the RNA analysis, RNA is extracted by spin columns and the isolated RNA can be used for qRT-PCR analysis. For the protein analysis, cells are labeled with fluorescently labeled antibodies and analyzed with flow cytometry. Use of active anti-CSC compounds leads to decreased expression of “stemness” genes/proteins compared to the mock treatment.

The complete experimental setting of the SCESM model from the seeding of the spheroids to the addition of the tested compounds and the final results readout is summarized in Figure 7.

## 4. Discussion

In this work, we developed a new cancer stem cell-enriched spheroid model (SCESM), which is faster than the traditional culture of free-floating spheres and enables higher CSC enrichment than the often used MCTS model. Due to the microplate format, a simple handling protocol, compatibility with multimodal detection systems, and sufficient detection signal, i.e., high stem marker expression, SCESM is suitable for HTS analyses of anti-CSC compounds.

To best recapitulate the complexity and heterogeneity of the primary tumor conditions, it would be reasonable to use primary cells for designing SCESM [32]. However, primary cell cultures have several drawbacks limiting their use for HTS; they are difficult to obtain, and their propagation is expensive and relatively limited. Additionally, they have high intra- and inter-sample variability, making comparisons between research groups difficult [32]. Hence, immortalized cell lines enabling reliable cell propagation and high uniformity of experimental conditions are more suitable for HTS applications. The FaDu cells used in this work are particularly suitable for a cancer stem-like model as they already express high basic levels of several stem markers and are known to form compact spheroids [28,39,40].

To imitate drug penetration and response in real tumors, non-adherent 3-D models proved superior to standard adherent 2-D models [25,41,42]. Particularly, 3-D spheroid models gained much popularity in studying CSC in recent years [18,20]. The two most popular spheroid growing techniques are cultures of free-floating spheres (i.e., tumorsphere model and spheroid forming assay) and MCTS [8,12]. Cultures of free-floating spheres were applied to isolate CSC from a variety of tumors, including brain, breast, lung, and HNSCC [12,43,44]. This is an important model for unraveling CSC characteristics [45] and has also been used to study the effect of anticancer agents like cetuximab, quercetin, resveratrol, and others [46,47,48,49]. However, this technique demands prolonged cultivation (>2 weeks) with many manipulation steps. Additionally, as it employs a flat ULA surface, it generates multiple spheroids highly diverse in size, with no single spheroid spatial separation. Hence, the culture of free-floating spheres is poorly compatible with HTS demands [17]. The MCTS model, on the other hand, is designed in microtiter formats, has shorter cultivation times, is compatible with automated and multimodal detection systems, and is thus often employed in the search for anticancer compounds using HTS [18,50]. Many anticancer compounds have been analyzed using MCTS, including a dual phosphoinositide 3-kinase (PI3K) and the mammalian target of rapamycin (mTOR) inhibitor dactolisib, a tyrosine kinase inhibitor sunitinib, and others [15,17]. However, as MCTS employs FBS-supplemented medium with a known differentiation effect, only partial enrichment of CSC is possible. Therefore, the effect of anti-CSC compounds in MCTS is hard to detect [21,22,32]. To circumvent the differentiation effect of FBS-supplemented media, variations of MCTS are being developed. Recently, glucose deprivation led to MCTS with inner hypoxia [51], while two other studies used variations of serum-free media with Y-27632 or insulin-hydrocortisone supplementation and displayed CSC enrichment [52,53].

In our study, we combined positive properties of both most commonly used spheroid growing techniques, i.e., culture of free-floating spheres and MCTS. Similar to conditions for cultures of free-floating spheres, we used a complete stem medium. Its specific composition of growth factors (basic fibroblast growth factor and epidermal growth factor), hormones (insulin and progesterone), and without serum supplementation, the stem medium is an important determinant supporting the growth and proliferation of CSCs [12,32,54]. The use of stem medium enabled a significantly higher expression of seven out of eight measured stem markers in SCESM spheroids compared to the MCTS in the same short cultivation time.

When testing the effects of the stem medium on spheroid growth, the SCESM spheroids also displayed a significantly faster diameter increase compared to the MCTS. This is consistent with the mitogenic effect of the two growth factors (EGF, bFGF) added to the stem medium. [32]. Interestingly though, EGF is also a known epithelial differentiation factor; however, this effect seems to be successfully counteracted by other components of the stem medium as evident by the expression profiles of the SCESM spheroids [55]. The SCESM spheroids exceeded 450 µm already at day 3 and 800 µm at day four (these diameters enabling the development of hypoxia and necrotic core, respectively) [28,56,57], both stimulating the dedifferentiation process and CSC enrichment [30,58,59,60]. Additionally, analyses of the proliferation marker Ki-67 and the 7-AAD nuclear stain suggested that only the outermost rim of cells is fast proliferating, while the cells deeper in are poorly/not proliferating. Such a result is consistent with other studies on the proliferation of spheroids, which have shown that live cells are present only in the outer layer of a spheroid, with fast proliferating cells present only in the outer narrow rim of the spheroid live cell area [28,56]. This result further suggests the presence of CSC in the hypoxia-developing depths of SCESM spheroids. The confocal analysis showed the absence of a signal inside the spheroid, which could indicate the presence of a hole inside the spheroid, as already reported in similarly large spheroids [28], or the incomplete penetration of dyes into the interior of the spheroid. Nevertheless, since stain control traveled deeper into the spheroid than the studied Ki-67 dye, we believe the results of proliferation marker staining represent the true image of proliferating cells in the spheroid.

Like in the MCTS model, we seeded a high concentration of cells in round-bottom ULA wells, with a subsequent centrifugation step immediately after seeding. Using ULA plates prevents the cells from adhering to the surface and therefore they remain in suspension. The centrifugation step enables the formation of a uniform cell mass and the formation of a single spheroid per well, which allows for the addition of a single active ingredient to an individual spheroid. Optimal conditions for a rapid formation of large, compact, uniform, and spatially separated spheroids with nutrient gradient/hypoxia development were set at the seeding of 3500 cells/well and cultivation of seven days. The compact spheroids, for which FaDu cells are known [28], enable an even intake of nutrients across the spheroid surface and easy handling as they are quite resistant to shear stress during manipulation. The uniformity of the spheroids is also important, as it allows the same diffusion conditions for all compounds tested. At day seven, the SCESM spheroid size stabilized. The result is in line with previous reports showing a plateau phase of spheroid growth [61]. In this, the spheroids’ growth pattern is similar to that of solid tumors, with an initial exponential growth period (i.e., the avascular growth phase), followed by a dormant phase during which the tumors develop angiogenic, invasive, and metastasizing properties characteristic of CSC [11,62]. A stable spheroid size is also important in models for testing anti-CSC agents since changes in spheroid size during treatment can be directly attributed to the effect of the active substance studied.

A sufficient detection signal enabling a good signal to noise ratio is also a prerequisite for HTS [63]. Recently, Spina et al. developed a drug screening system for the identification of CSC differentiation-inducing agents, employing FBS-supplemented MCTS and a lentiviral transcriptional green fluorescent protein (GFP) reporter system [64]. While the GFP reporter enables a strong and easy readout, it allows the monitoring of only a single differentiation marker for a single cell type. Tracking the reduction of stem marker expression instead would offer more universality, though unfortunately, no universal stem marker exists for the identification of CSC. This can be improved by using multiple markers instead of one [11]. Alternatively, functional assays, such as the limiting dilution assay, usually used to confirm the CSC properties of primary culture cells, can be employed. Unfortunately, in immortalized cell lines like FaDu, proliferation assays do not seem reasonable, since immortalized cell lines are characterized by unlimited proliferation [65]. For SCESM, we defined CD44, CD73, CD90, and CD133 as a set of stem markers increased at both the transcriptional and the protein level, enabling the tracking of CSC enrichment at both levels. Thus, depending on the design of compound screening, mRNA analysis with standardized protocols (but with a requirement for real-time expression analysis equipment) or protein analysis (requiring several optimization steps and use of flow cytometry or confocal microscopy) can be performed (Figure 7). To exemplify the usefulness of the SCESM and markers selected, we used ATRA, a known differentiating agent used also in HNSCC therapy [23,24]. As expected, treating SCESM with ATRA resulted in strongly reduced expression of stem markers, confirming both our choice of readout markers and the usefulness of SCESM for anti-CSC active substance screening. These results are even further validated by a strong increase of the IVL epithelial differentiation marker.

Last but not least, to enable the screening of large compound collections, HTS must be cost-effective [63]. Although the cost of seeding and cultivating the SCESM is slightly higher compared to the MCTS method, this is balanced out with a much higher CSC enrichment and thus a better readout. On the other hand, with regard to the consumption of reagents, SCESM’s seeding and 7-day cultivation are much more favorable to the 2–3-week cultivation for cultures of free-floating spheres.

Altogether, our cancer stem-cell enrichment model is a compromise between tumor complexity and protocol simplicity. It is an example of a simple spheroid model formed by only one cell type. Although it does not capture the complexity of the real tumor, it provides high CSC enrichment and is therefore suitable only for testing anti-CSC active substances but not active substances targeting other tumor cells, usually present in complex tumors. Due to SCESM’s simple establishment, uniformity, short cultivation time, and spatial separation, which allows for the addition of a single active ingredient to an individual spheroid, the model is well suited for the first HTS hit compounds identification, as also validated by the ATRA treatment. Such simple models enable cheaper first compound identification, narrowing further validation of the detected active substances in more complex and costly spheroidal or animal models [25].

## Figures and Tables

**Table 1:** Primer sequences.

Target	Forward Primer Sequence (5′–3′)	Reverse Primer Sequence (5′–3′)
*B2M*	TTCTGGCCTGGAGGCTATC	TCAGGAAATTTGACTTTCCATTC
*ALDH1A1*	GCACGCCAGACTTACCTGTC	AGCATCCATAGTACGCCACG
*CD44*	GAAGAAGGTGTGGGCAGAAG	TCTGCAGGTTCCTTGTCTCA
*PROM1*	TAATGAGCATACTGGAAGCA	CTGCACCCAACAGAAAGAT
*POU5F1*	CGAAAGAGAAAGCGAACCAG	ATCTGCTGCAGTGTGGGTTT
*SOX2*	GCCTGGGCGCCGAGTGGA	GGGCGAGCCGTTCATGTAGGTCTG
*NANOG*	GATGCAAGAACTCTCCAACATCC	TTGCTATTCTTCGGCCAGTTGT
*THY1*	CTAACAGTCTTGCAGGTCTCC	CTTCTTTGTCTCACGGGTCAG
*NT5E*	TCTCTCAAATCCAGGGACAAATT	GTCCACACCCCTCACTTTCT
*ENG*	GAGGCGGTGGTCAATATCCT	GGACACTCTGACCTGCACAA
*IVL*	GCCTTACTGTGAGTCTGGTTGA	GGAGGAACAGTCTTGAGGAGC
*CDH1*	GAGGGGTTAAGCACAACAGC	ACGACGTTAGCCTCGTTCTC

## Supplementary Materials

The following are available online at https://www.mdpi.com/2073-4409/9/7/1707/s1, Figure S1: Relative gene expression of stem markers during optimization of differentiation treatment.

## References

[1] Shah A., Patel S., Pathak J., Swain N., Kumar S. (2014). The Evolving Concepts of Cancer Stem Cells in Head and Neck Squamous Cell Carcinoma. Sci. World J..

[2] Bray F., Ferlay J., Soerjomataram I., Siegel R.L., Torre L.A., Jemal A. (2018). Global cancer statistics 2018: GLOBOCAN estimates of incidence and mortality worldwide for 36 cancers in 185 countries. CA Cancer J. Clin..

[3] Kaseb H.O., Fohrer-Ting H., Lewis D.W., Lagasse E., Gollin S. (2016). Identification, expansion and characterization of cancer cells with stem cell properties from head and neck squamous cell carcinomas. Exp. Cell Res..

[4] Gao W., Wu D., Wang Y., Wang Z., Zou C., Dai Y., Leung A.K., Teoh J.Y.-C., Chan F.L. (2018). Development of a novel and economical agar-based non-adherent three-dimensional culture method for enrichment of cancer stem-like cells. Stem Cell Res. Ther..

[5] Vermeulen L., Melo F.D.S.E., Richel D.J., Medema J.P. (2012). The developing cancer stem-cell model: Clinical challenges and opportunities. Lancet Oncol..

[6] Carnero A., Mayea Y.G., Mir C., Lorente J., Rubio I., Lleonart M.E. (2016). The cancer stem-cell signaling network and resistance to therapy. Cancer Treat. Rev..

[7] Konrad C.V., Murali R., Varghese B.A., Nair R. (2017). The role of cancer stem cells in tumor heterogeneity and resistance to therapy. Can. J. Physiol. Pharmacol..

[8] Franco S.S., Szczesna K., Iliou M.S., Al-Qahtani M.H., Mobasheri A., Kobolak J., Dinnyes A. (2016). In vitro models of cancer stem cells and clinical applications. BMC Cancer.

[9] Gupta P.B., Önder T.T., Jiang G., Tao K., Kuperwasser C., A Weinberg R., Lander E.S. (2009). Identification of selective inhibitors of cancer stem cells by high-throughput screening. Cell.

[10] Al-Hajj M., Wicha M.S., Benito-Hernandez A., Morrison S.J., Clarke M.F. (2003). Prospective identification of tumorigenic breast cancer cells. Proc. Natl. Acad. Sci. USA.

[11] Peitzsch C., Nathansen J., Schniewind S.I., Schwarz F., Dubrovska A. (2019). Cancer Stem Cells in Head and Neck Squamous Cell Carcinoma: Identification, Characterization and Clinical Implications. Cancers.

[12] Weiswald L.-B., Bellet D., Dangles-Marie V. (2015). Spherical Cancer Models in Tumor Biology. Neoplasia.

[13] Harper L.J., Piper K., Common J.E., Fortune F., MacKenzie I.C. (2007). Stem cell patterns in cell lines derived from head and neck squamous cell carcinoma. J. Oral Pathol. Med..

[14] Zhang Q., Shi S., Yen Y., Brown J., Ta J.Q., Le A.D. (2010). A subpopulation of CD133+ cancer stem-like cells characterized in human oral squamous cell carcinoma confer resistance to chemotherapy. Cancer Lett..

[15] Kochanek S.J., Close D.A., Camarco D.P., Johnston P.A. (2020). Maximizing the Value of Cancer Drug Screening in Multicellular Tumor Spheroid Cultures: A Case Study in Five Head and Neck Squamous Cell Carcinoma Cell Lines. SLAS Discov. Adv. Life Sci. R&D.

[16] Roy S., Roy S., Kar M., Padhi S., Saha A., Anuja K., Banerjee B. (2018). Role of p38 MAPK in disease relapse and therapeutic resistance by maintenance of cancer stem cells in head and neck squamous cell carcinoma. J. Oral Pathol. Med..

[17] Close D.A., Camarco D.P., Shan F., Kochanek S.J., Johnston P. (2017). The Generation of Three-Dimensional Head and Neck Cancer Models for Drug Discovery in 384-Well Ultra-Low Attachment Microplates. Adv. Struct. Saf. Stud..

[18] Fang Y., Eglen R.M. (2017). Three-Dimensional Cell Cultures in Drug Discovery and Development. SLAS Discov. Adv. Life Sci. R&D.

[19] Tekkatte C., Gunasingh G.P., Cherian K.M., Sankaranarayanan K. (2011). “Humanized” Stem Cell Culture Techniques: The Animal Serum Controversy. Stem Cells Int..

[20] Ishiguro T., Ohata H., Sato A., Yamawaki K., Enomoto T., Okamoto K. (2017). Tumor-derived spheroids: Relevance to cancer stem cells and clinical applications. Cancer Sci..

[21] Lee J., Kotliarova S., Kotliarov Y., Li A., Su Q., Donin N.M., Pastorino S., Purow B.W., Christopher N., Zhang W. (2006). Tumor stem cells derived from glioblastomas cultured in bFGF and EGF more closely mirror the phenotype and genotype of primary tumors than do serum-cultured cell lines. Cancer Cell.

[22] Ricci-Vitiani L., Lombardi D.G., Pilozzi E., Biffoni M., Todaro M., Peschle C., de Maria R. (2006). Identification and expansion of human colon-cancer-initiating cells. Nature.

[23] Guruvayoorappan C., Grace V.B. (2010). All Trans Retinoic Acid and Cancer. Immunopharmacol. Immunotoxicol..

[24] Khuri F.R., Lee J.J., Lippman S.M., Kim E.S., Cooper J.S., Benner S.E., Winn R., Pajak T.F., Williams B., Shenouda G. (2006). Randomized Phase III Trial of Low-dose Isotretinoin for Prevention of Second Primary Tumors in Stage I and II Head and Neck Cancer Patients. J. Natl. Cancer Inst..

[25] Shan F., Close D.A., Camarco D.P., Johnston P. (2018). High-Content Screening Comparison of Cancer Drug Accumulation and Distribution in Two-Dimensional and Three-Dimensional Culture Models of Head and Neck Cancer. ASSAY Drug Dev. Technol..

[26] Zhu M., Zhang C., Chen D., Chen S., Zheng H. (2019). MicroRNA-98-HMGA2-POSTN signal pathway reverses epithelial-to-mesenchymal transition in laryngeal squamous cell carcinoma. Biomed. Pharmacother..

[27] Gole B., Mian E., Rall M., Wiesmüller L. (2017). Base excision repair proteins couple activation-induced cytidine deaminase and endonuclease G during replication stress-induced MLL destabilization. Leukemia.

[28] Kochanek S.J., Close D.A., Johnston P. (2019). High Content Screening Characterization of Head and Neck Squamous Cell Carcinoma Multicellular Tumor Spheroid Cultures Generated in 384-Well Ultra-Low Attachment Plates to Screen for Better Cancer Drug Leads. ASSAY Drug Dev. Technol..

[29] Riffle S., Pandey R.N., Albert M., Hegde R.S. (2017). Linking hypoxia, DNA damage and proliferation in multicellular tumor spheroids. BMC Cancer.

[30] Riffle S., Hegde R.S. (2017). Modeling tumor cell adaptations to hypoxia in multicellular tumor spheroids. J. Exp. Clin. Cancer Res..

[31] Wan A.C. (2019). Primitive Cancer Cell States: A Target for Drug Screening?. Trends Pharmacol. Sci..

[32] Bielecka Z.F., Maliszewska-Olejniczak K., Safir I.J., Szczylik C., Czarnecka A.M. (2016). Three-dimensional cell culture model utilization in cancer stem cell research. Boil. Rev..

[33] Yu S.S., Cirillo N. (2019). The molecular markers of cancer stem cells in head and neck tumors. J. Cell. Physiol..

[34] van Schaijik B., Davis P.F., Wickremesekera A.C., Tan S.T., Itinteang T. (2017). Subcellular localisation of the stem cell markers OCT4, SOX2, NANOG, KLF4 and c-MYC in cancer: A review. J. Clin. Pathol..

[35] Pazhanisamy S. (2013). Adult Stem Cell and Embryonic Stem Cell Markers. Mater. Methods.

[36] Dominici M., le Blanc K., Mueller I., Slaper-Cortenbach I., Marini F., Krause D., Deans R., Keating A., Prockop D., Horwitz E. (2006). Minimal criteria for defining multipotent mesenchymal stromal cells. The International Society for Cellular Therapy position statement. Cytotherapy.

[37] Yoshida S., Yasuda M., Miyashita H., Ogawa Y., Yoshida T., Matsuzaki Y., Tsubota K., Okano H., Shimmura S. (2011). Generation of Stratified Squamous Epithelial Progenitor Cells from Mouse Induced Pluripotent Stem Cells. PLoS ONE.

[38] Gall T.M.H., Frampton A.E. (2013). Gene of the month: E-cadherin (CDH1). J. Clin. Pathol..

[39] Fukusumi T., Ishii H., Konno M., Yasui T., Nakahara S., Takenaka Y., Yamamoto Y., Nishikawa S., Kano Y., Ogawa H. (2014). CD10 as a novel marker of therapeutic resistance and cancer stem cells in head and neck squamous cell carcinoma. Br. J. Cancer.

[40] Fík Z., Dvořánková B., Kodet O., Bouček J., Betka J.A., André S., Gabius H.-J., Šnajdr P., Smetana K., Chovanec M. (2014). Towards dissecting molecular routes of intercellular communication in the tumour microenvironment: Phenotypic plasticity of stem cell-associated markers in co-culture (carcinoma cell/fibroblast) systems. Folia Boil..

[41] Ryan S.-L., Baird A.-M., Vaz G., Urquhart A.J., Senge H., Richard D.J., O’Byrne K., Davies A.M., Senge M.O. (2016). Drug Discovery Approaches Utilizing Three-Dimensional Cell Culture. ASSAY Drug Dev. Technol..

[42] Ham S.L., Joshi R., Thakuri P.S., Tavana H. (2016). Liquid-based three-dimensional tumor models for cancer research and drug discovery. Exp. Boil. Med..

[43] Singh S.K., Clarke I.D., Terasaki M., Bonn V.E., Hawkins C., Squire J., Dirks P.B. (2003). Identification of a cancer stem cell in human brain tumors. Cancer Res..

[44] Chiou S.-H., Yu C.-C., Huang C.-Y., Lin S.-C., Liu C.-J., Tsai T.-H., Chou S.-H., Chien C.-S., Ku H.H., Lo J.-F. (2008). Positive Correlations of Oct-4 and Nanog in Oral Cancer Stem-Like Cells and High-Grade Oral Squamous Cell Carcinoma. Clin. Cancer Res..

[45] Muñoz-Galván S., Felipe-Abrio B., Verdugo-Sivianes E.M., Perez M., Jiménez-García M.P., Suarez-Martinez E., Estevez-Garcia P., Carnero A. (2020). Downregulation of MYPT1 increases tumor resistance in ovarian cancer by targeting the Hippo pathway and increasing the stemness. Mol. Cancer.

[46] Lee C.-H., Yu C.-C., Wang B.-Y., Chang W.-W. (2015). Tumorsphere as an effective in vitro platform for screening anti-cancer stem cell drugs. Oncotarget.

[47] Chang W.-W., Hu F.-W., Yu C.-C., Wang H.-H., Feng H.-P., Lan C., Tsai L.-L., Chang Y.-C. (2012). Quercetin in elimination of tumor initiating stem-like and mesenchymal transformation property in head and neck cancer. Head Neck.

[48] Hu F.-W., Tsai L.-L., Yu C.-H., Chen P.-N., Chou M.-Y., Yu C.-C. (2012). Impairment of tumor-initiating stem-like property and reversal of epithelial-mesenchymal transdifferentiation in head and neck cancer by resveratrol treatment. Mol. Nutr. Food Res..

[49] Zscheppang K., Kurth I., Wachtel N., Dubrovska A., Kunz-Schughart L.A., Cordes N. (2016). Efficacy of Beta1 Integrin and EGFR Targeting in Sphere-Forming Human Head and Neck Cancer Cells. J. Cancer.

[50] LaBarbera D.V., Reid B.G., Yoo B.H. (2012). The multicellular tumor spheroid model for high-throughput cancer drug discovery. Expert Opin. Drug Discov..

[51] Senkowski W., Zhang X., Olofsson M.H., Isacson R., Höglund U., Gustafsson M., Nygren P., Linder S., Larsson R., Fryknäs M. (2015). Three-Dimensional Cell Culture-Based Screening Identifies the Anthelmintic Drug Nitazoxanide as a Candidate for Treatment of Colorectal Cancer. Mol. Cancer Ther..

[52] Guo X., Chen Y., Ji W., Chen X., Li C., Ge R. (2018). Enrichment of cancer stem cells by agarose multi-well dishes and 3D spheroid culture. Cell Tissue Res..

[53] Herheliuk T., Perepelytsina O., Ugnivenko A., Ostapchenko L., Sydorenko M. (2019). Investigation of multicellular tumor spheroids enriched for a cancer stem cell phenotype. Stem Cell Investig..

[54] Zhang G., Xie Y.-K., Miao X.-B., Ma L., Jin C. (2012). Esophageal cancer tumorspheres involve cancer stem-like populations with elevated aldehyde dehydrogenase enzymatic activity. Mol. Med. Rep..

[55] Herbst R.S. (2004). Review of epidermal growth factor receptor biology. Int. J. Radiat. Oncol..

[56] Grimes D.R., Kelly C., Bloch K., Partridge M. (2014). A method for estimating the oxygen consumption rate in multicellular tumour spheroids. J. R. Soc. Interf..

[57] Sant S., Johnston P. (2017). The production of 3D tumor spheroids for cancer drug discovery. Drug Discov. Today Technol..

[58] Wang P., Wan W.-W., Xiong S.-L., Feng H., Wu N. (2017). Cancer stem-like cells can be induced through dedifferentiation under hypoxic conditions in glioma, hepatoma and lung cancer. Cell Death Discov..

[59] He J., Xiong L., Li Q., Lin L., Miao X., Yan S., Hong Z., Yang L., Wen Y., Deng X. (2017). 3D modeling of cancer stem cell niche. Oncotarget.

[60] Carnero A., Lleonart M. (2016). The hypoxic microenvironment: A determinant of cancer stem cell evolution. BioEssays.

[61] Costa E.C., Moreira A.F., de Melo-Diogo D., Gaspar V.M., Carvalho M.P., Correia I.J. (2016). 3D tumor spheroids: An overview on the tools and techniques used for their analysis. Biotechnol. Adv..

[62] Song J., Chang I., Chen Z., Kang M., Wang C.-Y. (2010). Characterization of Side Populations in HNSCC: Highly Invasive, Chemoresistant and Abnormal Wnt Signaling. PLoS ONE.

[63] Powell D.J., Hertzberg R.P., Macarrόn R. (2016). Design and Implementation of High-Throughput Screening Assays. Adv. Struct. Saf. Stud..

[64] Spina R., Voss D.M., Asnaghi L., Sloan A., Bar E.E. (2018). Flow Cytometry-based Drug Screening System for the Identification of Small Molecules That Promote Cellular Differentiation of Glioblastoma Stem Cells. J. Vis. Exp..

[65] Verma A., Ashish S., Verma A.S. (2014). Animal Biotechnology. Chapter 12—Animal Tissue Culture: Principles and Applications.

