# Supplementary material for: Head and Neck Cancer Stem Cell-Enriched Spheroid Model for Anticancer Compound Screening
Source: Cells. 2020 Jul 16;9(7):1707. doi: 10.3390/cells9071707 (PMC7408407; doi:10.3390/cells9071707)
Supplement: Supplementary file 1 [file cells-09-01707-s001.pdf]

Supplementary Materials

Supplementary Figure 1

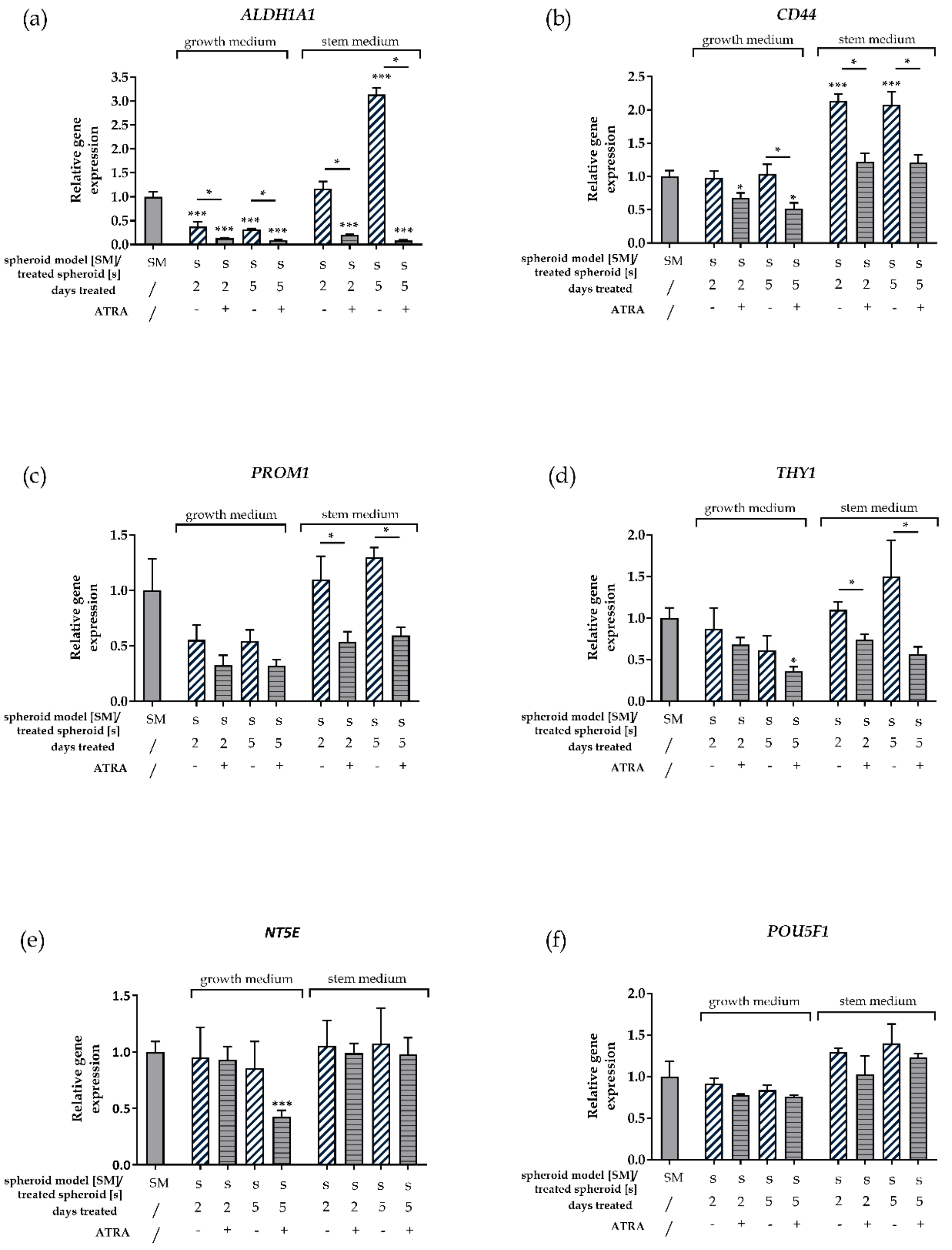

Figure S1. Relative gene expression of stem markers during optimization of differentiation treatment. SCESM spheroids were treated with two different media (growth medium ± ATRA, stem medium ± ATRA) and two different treatment durations (2 and 5 days). Relative gene expression of six stem markers was assessed in untreated 7- days old SCESM spheroids and treated SCESM spheroids. (a) Relative gene expression of *ALDH1A1*; (b) Relative gene expression of *CD44*; (c) Relative gene expression of *PROM1*; (d) Relative gene expression of *THY1*; (e) Relative gene expression of *NT5E*; (f) Relative gene expression of *POU5F1.* Normalized mean values ± SE (n ≥ 5) are presented Asterisks above the graph bars represent the results of statistical analysis of the studied group vs. the control group - SM (*$p < 0.05$, ***$p < 0.001$). SM: 7 days old SCESM spheroids; s: treated SCESM spheroids;). - : control/ATRA untreated group; + : ATRA treated group.
